# High Fidelity Processing and Activation of the Human α-Defensin HNP1 Precursor by Neutrophil Elastase and Proteinase 3

**DOI:** 10.1371/journal.pone.0032469

**Published:** 2012-03-20

**Authors:** Prasad Tongaonkar, Amir E. Golji, Patti Tran, André J. Ouellette, Michael E. Selsted

**Affiliations:** 1 Department of Pathology and Laboratory Medicine, Keck School of Medicine, University of Southern California, Los Angeles, California, United States of America; 2 Kenneth Norris Comprehensive Cancer Center, University of Southern California, Los Angeles, California, United States of America; University of Tübingen, Germany

## Abstract

The azurophilic granules of human neutrophils contain four α-defensins called human neutrophil peptides (HNPs 1–4). HNPs are tridisulfide-linked antimicrobial peptides involved in the intracellular killing of organisms phagocytosed by neutrophils. The peptides are produced as inactive precursors (proHNPs) which are processed to active microbicides by as yet unidentified convertases. ProHNP1 was expressed in *E. coli* and the affinity-purified propeptide isolated as two species, one containing mature HNP1 sequence with native disulfide linkages (“folded proHNP1”) and the other containing non-native disulfide linked proHNP1 conformers (misfolded proHNP1). Native HNP1, liberated by CNBr treatment of folded proHNP1, was microbicidal against *Staphylococcus aureus*, but the peptide derived from misfolded proHNP1 was inactive. We hypothesized that neutrophil elastase (NE), proteinase 3 (PR3) or cathepsin G (CG), serine proteases that co-localize with HNPs in azurophil granules, are proHNP1 activating convertases. Folded proHNP1 was converted to mature HNP1 by both NE and PR3, but CG generated an HNP1 variant with an N-terminal dipeptide extension. NE and PR3 cleaved folded proHNP1 to produce a peptide indistinguishable from native HNP1 purified from neutrophils, and the microbicidal activities of *in vitro* derived and natural HNP1 peptides were equivalent. In contrast, misfolded proHNP1 conformers were degraded extensively under the same conditions. Thus, NE and PR3 possess proHNP1 convertase activity that requires the presence of the native HNP1 disulfide motif for high fidelity activation of the precursor *in vitro*.

## Introduction

Antimicrobial peptides (AMPs) produced by animal cells provide a first line of defense against potentially infectious microorganisms. In mammals, defensins and cathelicidins are the primary AMPs expressed in professional phagocytes such as neutrophils and macrophages [1]. Mammalian defensins comprise three structural subfamilies termed α-, β- and θ-defensins. While all defensins are arginine rich, tridisulfide containing peptides, they are distinguished from each other by the presence of unique disulfide motifs [2]. In addition, θ-defensins, which are approximately half the size of α- and β-defensins, are macrocyclic molecules which have only been isolated from leukocytes of Old World monkeys (reviewed in [2], [3]). Defensins have antimicrobial activities against diverse microbial targets that include bacteria [4], fungi [5], [6], viruses [7], [8], [9], and protozoa [10]. Certain α-, β-, and θ-defensins function as regulators of adaptive immune cells and thus function to modulate inflammation [11], [12], [13] and bridge innate and adaptive immunity [14].

In humans, four α-defensins, human neutrophil peptides 1–4 (HNP 1–4), are expressed in neutrophils and monocytes of myeloid lineage, and α-defensins (HD5 and HD6) are produced primarily in Paneth cells of the small intestine. In neutrophils, HNP 1–4 are stored in the cytoplasmic azurophil granules which are mobilized during phagocytosis resulting in the exposure of ingested microorganisms to high concentrations of HNPs in the phagolysosomes [2]. In the setting of bacteremic sepsis, HNPs also accumulate in the extracellular compartment [15], [16]. Several studies have demonstrated an extracellular role for myeloid α-defensins in regulating cellular activities including cell proliferation [17], [18], [19], neovascularization [20], [21], chemotaxis [22], and regulation of cytokines [23], [24] and signal transduction pathways [17], [19], [24], [25]. To date, these activities have been attributed to the mature, folded α-defensin.

Defensins of all three subfamilies are expressed as prepro-defensins containing an N-terminal signal peptide followed by the propeptide that contains the prosegment and the folded/oxidized mature peptide sequence [26]. The prosegment in α- and θ-defensin precursors is ∼45 amino acids in length, but that of β-defensin precursors is typically very short. In α-defensins, studies suggest that the anionic prosegment interacts with residues present in the mature peptide to maintain it in an inactive state until it reaches the correct cellular location, possibly to protect cellular compartments from peptide-mediated cytotoxic effects [27], [28], [29].

Processing of the human enteric α-defensin, HD5 occurs extracellularly and is mediated by trypsin [30], whereas mouse Paneth cell α-defensins are processed intracellularly by matrix metalloprotease 7 (MMP7, matrilysin) [31]. Neutrophil elastase (NE), an azurophil granule protease, was shown to correctly process RMAD4, a rhesus macaque neutrophil α-defensin *in vitro* [32]. Bovine and human cathelicidins, a distinct class of antimicrobial peptides, are processed by the azurophil granule proteases, NE [33] and proteinase 3 (PR3) [34] respectively. Studies with HL-60 cells, a human acute promyelocytic leukemia cell line, show that HNPs are biosynthesized and processed to mature peptides in these cells [35], but the proteases involved in maturation of human myeloid α-defensins have not been identified. Because HNPs co-localize to neutrophil granules containing the serine proteases NE, PR3 and cathepsin G (CG) [36], we hypothesized that HNP maturation is mediated by one or more of these proteinases. In this study, recombinant proHNP1 was used as substrate to analyze the human α-defensin convertase activities of NE, PR3, and CG. The requirement for the native HNP1 fold was analyzed by assessing the processing of folded and misfolded preparations of proHNP1. In addition, the antimicrobial activity of the resulting HNP-1 peptides was analyzed utilizing *Staphylococcus aureus* as the test organism.

## Results

### Expression and Purification of His_6_-proHNP1

**Figure 1:**
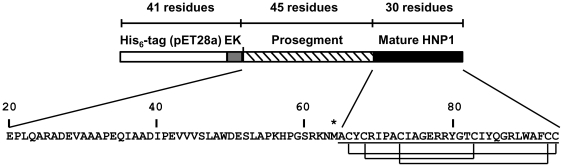
Schematic representation of His_6_-proHNP1. *Top Panel*. The 36 amino acid residue N-terminal extension showing the His_6_-tag from the pET28a+ vector followed by the enterokinase site and proHNP1 sequence in the His_6_-proHNP1 construct are shown. The numbers above indicate the number of amino acid residues in each segment. *Bottom Panel*. The proHNP1 sequence showing the mature sequence (underlined), the disulfide linkages between Cys residues, and the Met residue (indicated with an asterisk) at the junction of the pro- and mature sequence. The numbers indicate the residue number in the preproHNP1 precursor.

**Figure 2:**
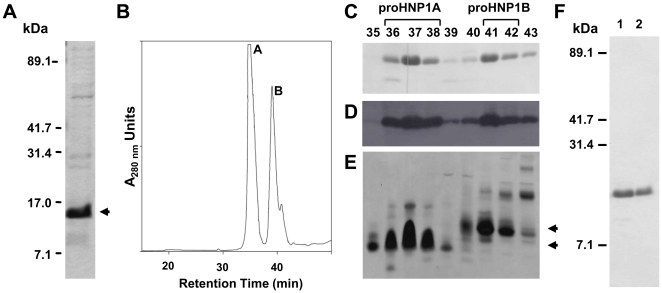
Purification of His_6_-proHNP1 foldamers. **A**) Ni-column eluant was analyzed by SDS-tricine PAGE and Coomassie stained; arrowhead indicates the major ∼12 kDa recombinant protein in the eluant. **B**) HPLC chromatogram of Ni-eluant resolved by semi-preparative C18 RP-HPLC generates two major peaks, A and B. **C**) HPLC fractions (numbers indicated) shown in panel B were resolved on SDS-tricine gel and stained with Coomassie blue, (**D**) analyzed by western blotting using anti-His_6_-tag antibody, and by (**E**) Coomassie staining following AU-PAGE. **F**) His_6_-proHNP1 containing fractions 36–38 (His_6_-proHNP1A) and fractions 41–42 (His_6_-proHNP1B) were pooled and further purified by RP-HPLC on C18 column and analyzed by Coomassie staining samples resolved on SDS-tricine gel (lane 1 - His_6_-proHNP1A, lane 2 - His_6_-proHNP1B).

Recombinant hexa-His-tagged pro-HNP-1 (Fig. 1) was expressed at approximately 2 mg/L in *E. coli* cells. The imidazole eluant from the nickel affinity column (Ni-eluant) contained a major band of ∼12 kDa, consistent with the molecular weight of His_6_-proHNP1 (theory  = 12647 A.M.U.; Fig. 2A). C18 reversed phase high performance liquid chromatography (RP-HPLC) of the Ni-eluant produced two major peaks (A and B; Fig. 2B). On reducing SDS-tricine PAGE, peaks A and B contained the ∼12 kDa band (Fig. 2C) and both were detected with anti-His_6_ antibody confirming the presence of His_6_-proHNP1 (Fig. 2D). On AU-PAGE, His_6_-proHNP1 present in peak A (proHNP1A) migrated more rapidly than that contained in peak B (proHNP1B; Fig. 2E), suggesting that proHNP1A and proHNP1B are differently folded forms of the recombinant protein. This was confirmed by the finding that DTT-reduced proHNP1A and proHNP1B co-eluted on RP-HPLC (data not shown) and by demonstrating that folding of proHNP1B in the presence of reduced/oxidized glutathione led to the formation of proHNP1A (Text S1 and Figure S1). Thus, proHNP1A and proHNP1B contain folded and misfolded His_6_-proHNP1 respectively. ProHNP1A and proHNP1B-containing fractions were pooled separately and purified to homogeneity by a second round of RP-HPLC (Fig. 2F).

### HNP1 derived from proHNP1A and proHNP1B

**Figure 3:**
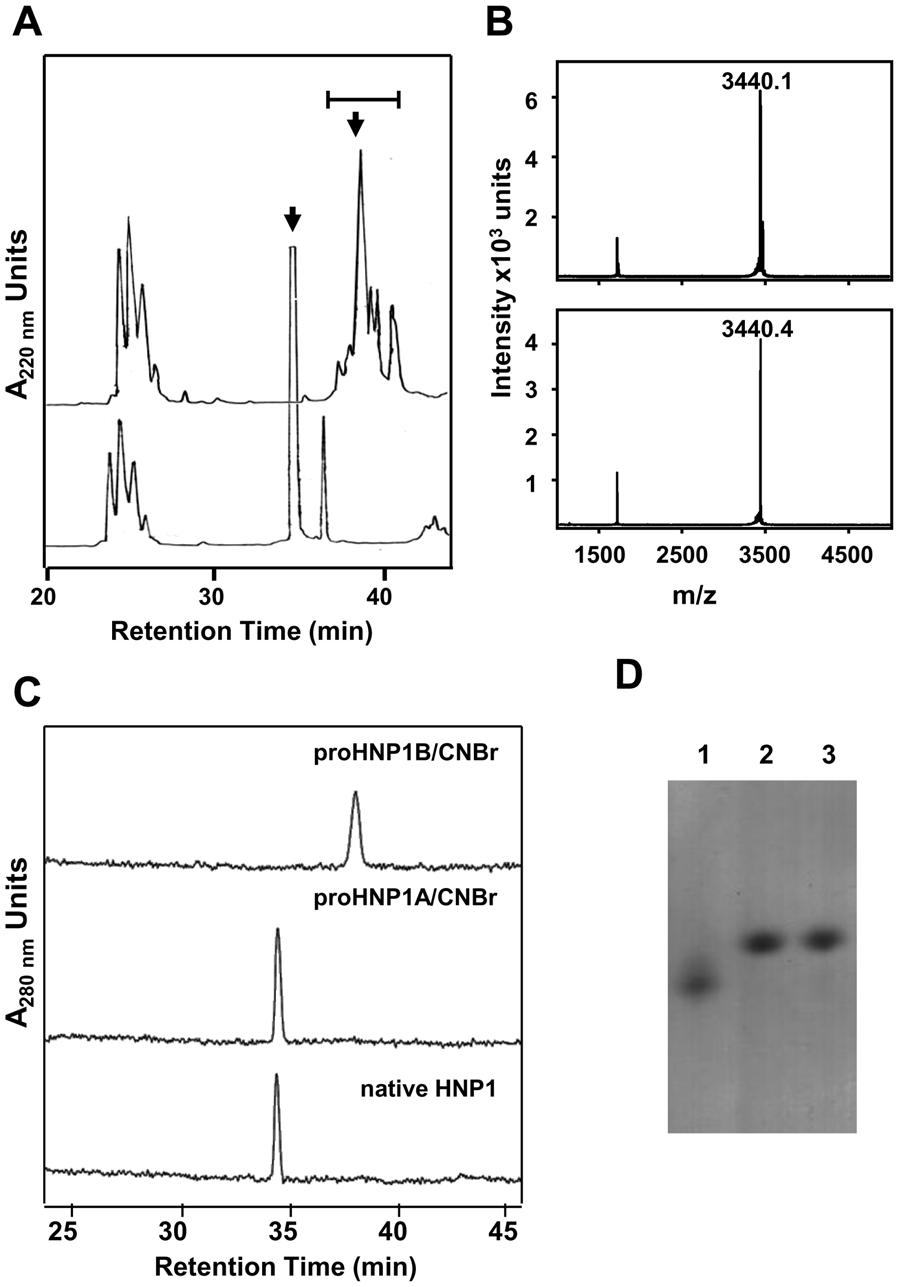
HNP1 forms released by CNBr cleavage of His_6_-proHNP1. HNP1 peptides were generated by CNBr cleavage of folded His_6_-proHNP1A and misfolded His_6_-proHNP1B. **A**) HNP1 generated by CNBr cleavage of His_6_-proHNP1B (upper tracing) and His_6_-proHNP1A (lower tracing) were resolved by C18 RP-HPLC. Bar indicates fractions containing peptides with molecular weight consistent with that of folded/oxidized HNP1 and peaks marked with arrows were further purified by HPLC. **B**) MALDI-TOF MS of CNBr-cleaved HNP1 derived from His_6_-proHNP1B (upper panel) and His_6_-proHNP1A (lower panel). **C**) Analytical C18 RP-HPLC using the Alliance system of HNP1 generated by CNBr-cleavage of His_6_-proHNP1B and His_6_-proHNP1A compared with neutrophil derived native HNP1. **D**) AU-PAGE analysis followed by Coomassie staining of 2 µg of each sample shown in panel C: peak B-derived HNP1 (lane 1), peak A-derived HNP1 (lane 2), native HNP1 (lane 3).

The presence of a Met residue at the junction of the prosegment and mature peptide in proHNP1 sequence (see Fig. 1) offered a non-enzymatic approach to generate HNP1 from proHNP1 utilizing cyanogen bromide (CNBr) cleavage [28]. Cleavage of proHNP1A with CNBr, followed by C18 RP-HPLC, gave rise to a major peptide product (Fig. 3A) with a mass (Fig. 3B) and HPLC retention time (Fig. 3C) matching that of natural HNP1. However, CNBr digestion of proHNP1B generated multiple species (Fig. 3A) that had masses consistent with oxidized HNP1 (Fig. 3B), but which eluted later than native HNP1 in RP-HPLC (Fig. 3C). On AU PAGE, HNP1 derived from proHNP1A comigrated with natural HNP1. However, HNP1 derived from proHNP1B migrated more rapidly than the natural standard (Fig. 3D). These data demonstrate that proHNP1A contained folded HNP1 with native disulfide linkages but the mature peptide in proHNP1B, although oxidized, is misfolded and contains non-native disulfide linkages. In solution, HNP1 exists as a non-covalent dimer [37] and the dimeric form corresponds to the single band observed in AU PAGE (Selsted *et al.*, unpublished data). Therefore, it is likely that the higher mobility of proHNP1B-derived HNP1 (Fig. 3D) is due to the monomeric state of this conformer.

### Microbicidal activities of HNP1 isoforms

**Figure 4:**
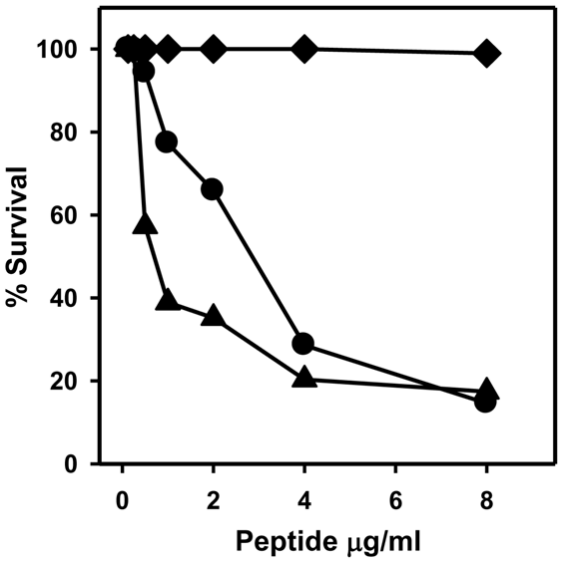
Staphylocidal activity of HNP1 generated by CNBr cleavage of His_6_-proHNP1. Microbicidal activity of native HNP1 (•) or HNP1-peptides generated by CNBr-cleavage of His_6_-proHNP1A (▴) and His_6_-proHNP1B (♦) was determined in a standard bactericidal assay against *S. aureus* 502a.

To analyze the microbicidal properties of the HNP1 conformers produced by CNBr cleavage of proHNP1A and proHNP1B, we tested the respective peptides for killing of *S. aureus in vitro*. HNP1 derived from proHNP1A had microbicidal activity similar to that of native HNP1 (Fig. 4). However, HNP1 derived from proHNP1B was devoid of staphylocidal activity (Fig. 4). Thus, formation of native disulfide linkages in HNP1 is essential for the staphylocidal activity of the peptide, and may reflect a further requirement for dimerization.

### Processing of His_6_-proHNP1 by azurophil granule proteases

**Figure 5:**
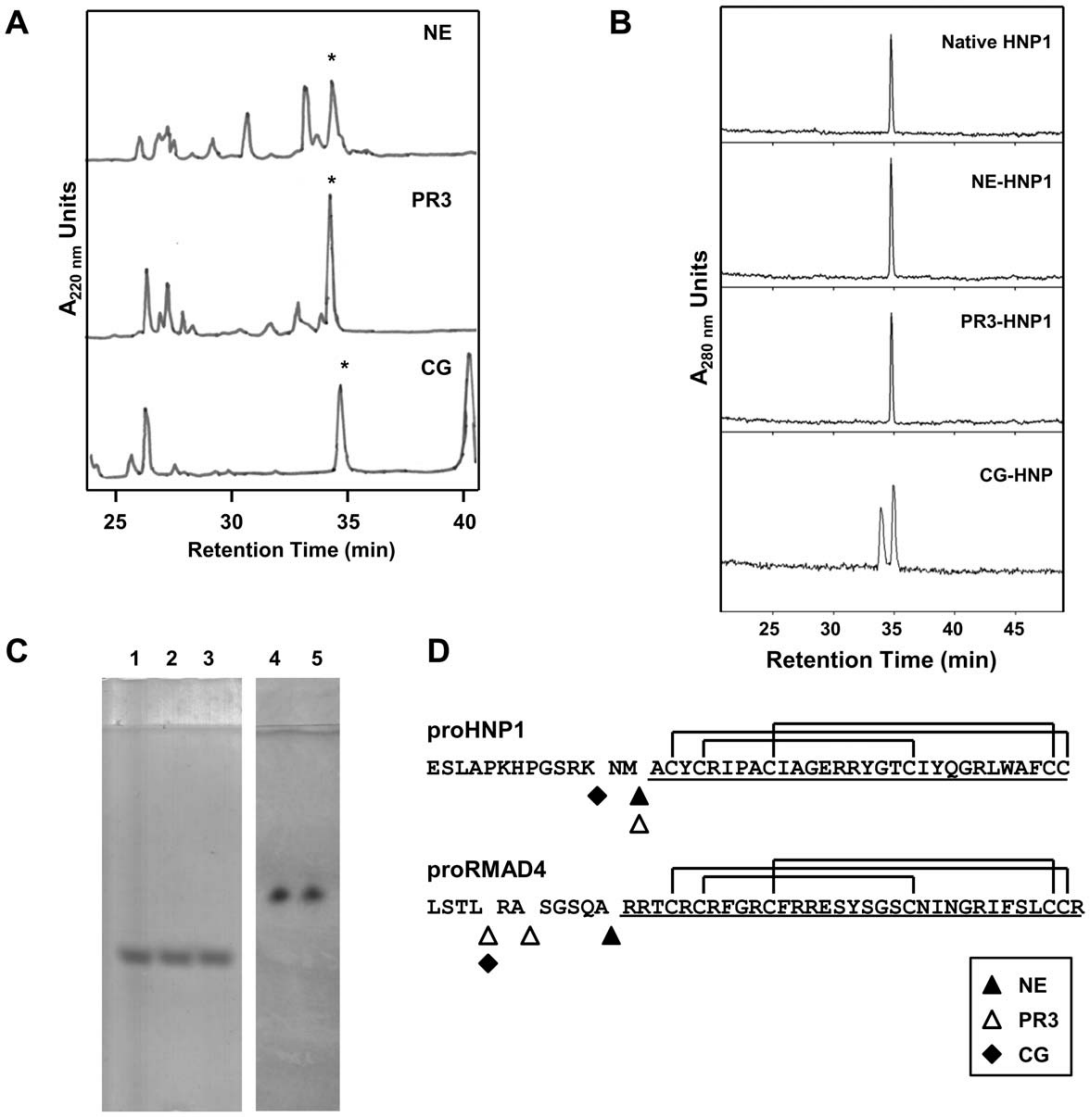
Proteolytic processing of His_6_-proHNP1 by azurophil granule serine proteases. **A**) His_6_-proHNP1A was digested with NE, PR3 and CG as described in Methods and resolved by C18 RP-HPLC. Peptides with masses matching that of HNP1 (asterisks) were identified by MALDI-TOF-MS. **B**) Analytical C18 RP-HPLC using the Alliance system of HNP1 isoforms derived from NE, PR3, and CG compared with native HNP1. **C**) AU-PAGE followed by Coomassie staining of native HNP1 (lanes 1 and 5) and HNP1 generated by processing with NE (lane 2), PR3 (lane 3) and CG (lane 4). **D**) The sequences of mature HNP1 and RMAD4 (underlined) and residues at the junction of their corresponding pro-segment are shown. ProHNP1 and proRMAD4 processing sites mediated by NE, PR3 and CG are indicated.

Previous studies have demonstrated that NE, PR3, and CG co-localize with HNPs in the azurophil granules of neutrophils. Kamdar *et al*. demonstrated that NE digestion of proRMAD4, precursor of rhesus myeloid α-defensin-4 (RMAD4), converted proRMAD4 to mature RMAD4. However, digestion of proRMAD4 with PR3 and CG generated RMAD4 variants with N-terminal extensions (Fig. 5D) [32]. To test for the role of these granule serine proteases in human HNP processing, proHNP1A was incubated in separate reactions with NE, PR3, or CG, and the RP-HPLC-purified cleavage products were analyzed by MALDI-TOF MS and analytical HPLC (Fig. 5 and Table 1). Incubation of His_6_-proHNP1 with NE or PR3 generated peptide products that co-eluted with native HNP1 in RP-HPLC (34.8 min; Fig. 5A, B) and had monoisotopic masses of 3440.6 and 3441.3 A.M.U. respectively, consistent with the mass of native HNP1 (3439.9 A.M.U. observed; theory = 3439.5 A.M.U.; Table 1). The identities of HNP1 derivatives were also confirmed by MALDI-TOF MS of each peptide following reduction and S-carboxyamidomethylation. In each case reduction and alkylation produced a mass increase of 348 A.M.U., confirming the presence of 6 disulfide-linked cysteine residues (Table 1). The NE- and PR3-derived peptides also co-migrated with native HNP1 on AU-gels (Fig. 5C; lanes 1–3). Thus, NE and PR3 processing of His_6_-proHNP1 generated a peptide that was indistinguishable from natural HNP1.

**Table 1:** Mass spectroscopic analysis of folded and reduced/alkylated HNP1^a^.

	Folded	S-CAM-derivative	Mass gain
Native HNP1	3439.9 [3439.5]	3787.8 [3788.7]	347.9 [348]
NE-derived HNP1	3440.6 [3439.5]	3789.7 [3788.7]	349.1 [348]
PR3-derived HNP1	3441.3 [3439.5]	3789.0 [3788.7]	347.7 [348]
CG- derived HNP1^b^	3685.4 [3685.6]	4033.3 [4033.8]	347.9 [348]

a Masses of natural and protease-derived HNP1 preparations were determined by MALDI-TOF MS before (Folded) and after reduction and S-carboxamidomethylation (S-CAM-derivative), and the observed gain in mass of the S-CAM product was calculated. All values shown are shown as atomic mass units (A.M.U.) of monoisotopic species; theoretical values are in brackets.

b mass of (Asn-Met)-HNP1.

Incubation of His_6_-proHNP1 with CG did not generate mature HNP1 but instead a digestion product that eluted from C18 HPLC later than HNP1 (Fig. 5A) and had a mass of 3685.4 A.M.U. This mass corresponds to HNP1 with a dipeptidyl (Asn-Met-) amino-terminal extension, consistent with Lys62↓Asn63 cleavage by CG (Fig. 5D). The CG liberated HNP1 product eluted as a single peak (Fig. 5A), which was resolved into two species by high resolution RP-HPLC (Alliance analytical HPLC system; Fig. 5B). Peptide in the second peak corresponded to the mass of Asn-Met-HNP-1 (3685.8 A.M.U.). Peptide in the earlier eluting peak was 3702.0 A.M.U., an increase of 16.2 A.M.U., corresponding to the sulfoxide derivative of methionine (theory = 16.0 A.M.U.) of Asn-Met-HNP1. Despite the chromatographic heterogeneity observed, CG-derived Asn-Met-HNP1 migrated as a single band on AU-PAGE, comigrating with native HNP1 (Fig. 5C, lanes 4 and 5).

### Analysis of Tag sequence effects on proHNP1 processing

Although the junctional sequences recognized by NE, PR3, and CG in His_6_-proHNP1 are identical to those in the natural precursor, we questioned whether the 41-residue extension of the proHNP-1 sequence resulting from the hexa-His and DDDDK tags and spacer sequences might alter protease interactions with the target. To test this, we produced proHNP1 without the N-terminal tag by incubating proHNP1A with enterokinase, thereby releasing the 41-residue tag sequence. This peptide was then treated with NE, PR3, or CG which liberated peptides with monoisotopic masses of 3439.7, 3439.7 and 3684.5 A.M.U., respectively, products equivalent to those generated from His_6_- proHNP1A. Thus the 41-residue N-terminal fusion tag did not alter the specificity of cleavage of proHNP1A by NE, PR3, or CG.

### Protease processing of misfolded His_6_-proHNP1

NE, PR3, and CG degraded proHNP1B, the misfolded form of the HNP1 precursor. In contrast to the discrete products generated by digestion of proHNP1A by the serine proteases (Fig. 5), NE, PR3, or CG treatment of proHNP1B yielded no mature HNP1 or HNP1 derivatives in the digestion mixtures detectable by C18 RP-HPLC or MALDI-TOF MS (data not shown). These results indicate that the correct fold and disulfide linkages in proHNP1 are required for processing of the precursor to mature peptide and to confer resistance to these activating convertases.

### Microbicidal activities of HNP1 products of proHNP1A processing by NE, PR3 and CG

**Figure 6:**
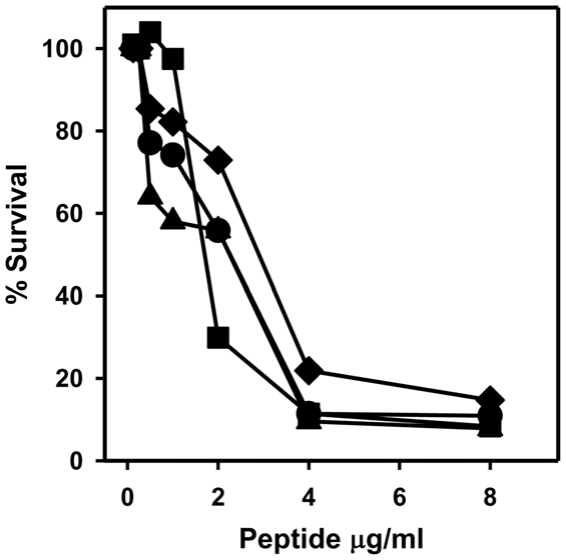
Microbicidal activity of HNP-peptides generated by NE, PR3 and CG processing of pro-HNP1. Bactericidal activity of HNP1 isoforms generated by processing with NE (▪), PR3 (▴), and CG (♦), with native HNP1 (•) was determined using *S. aureus* 502a as described in Methods.

The functional properties of HNP1 generated by serine protease processing of proHNP1A were tested in microbicidal assays against *S. aureus*. Natural HNP1 and NE, PR3 and CG processed preparations possessed nearly identical dose-dependent bactericidal activities against this organism (Fig. 6). These data demonstrate that HNP1 generated by NE and PR3 processing of proHNP1A is functionally equivalent to native HNP1. Of note, the Asn-Met-HNP1, produced by CG processing of proHNP1A, had staphylocidal activity equivalent to that of the fully processed HNP1 isoform.

## Discussion

To study the processing of human myeloid α-defensins, we established an *in vitro* assay utilizing recombinant proHNP1. Approximately 60% of the recombinant His_6_-proHNP1 was folded with native disulfide linkages (Fig. 2) and misfolded propeptide was readily converted to the folded form in buffer containing urea and reduced/oxidized glutathione (Fig. S1). The expression of folded and misfolded forms of proHNP1 allowed for the analysis of the structural requirements for HNP1-mediated bactericidal activity *in vitro*. CNBr treatment of folded (proHNP1A) or misfolded (proHNP1B) precursor generated different HNP1 conformers (Fig. 3). Despite the generation of oxidized peptides with masses matching that of natural HNP1, only the conformer generated by CNBr cleavage of proHNP1A had microbicidal activity against *S. aureus*. Processing of proHNP1A by NE or PR3 generated HNP1 peptide identical to natural HNP1, but proHNP1B was heterogeneous and was degraded by NE, PR3 and CG. These data demonstrate that the native HNP1 fold is necessary for proteolytic stability and accurate processing of the HNP1 propeptide by the three azurophil granule serine proteases investigated.

The requirement for the native HNP1 fold for microbicidal activity is consistent with a previous report demonstrating that an all Cys-to-Ala analog of HNP1 was nearly 10-fold less potent in bactericidal assays than the native peptide [38]. Similarly, disruption of disulfide linkages by mutagenesis of Cys residues to Ala or Ser in the human Paneth cell α-defensin HD5 reduced *in vitro* bactericidal activity demonstrating the requirement for the disulfides for *in vitro* bactericidal activity of this peptide [39]. In contrast, disruption of disulfide linkages in mouse enteric defensin, Crp4, by Cys-to-Ala mutagenesis did not eliminate microbicidal activities of the peptide [40].

Results presented here support the hypothesis that NE and PR3, proteases that co-localize with myeloid α-defensins, participate in the intracellular maturation of HNP1. In previous studies with HL-60 and chronic myelogenous leukemia cells, Valore and Ganz demonstrated sequential proteolytic processing of proHNP with the formation of a 56-residue intermediate that is subsequently processed to mature HNP. While proHNPs were found exclusively in the cytoplasmic/microsomal fraction, the granule fraction contained only the intermediate and mature HNP [35]. Therefore it is likely that proHNP-processing by NE and PR3 occurs during the packaging of HNPs in azurophil granules. CG processing of proHNP1A produced a peptide that was extended at the amino terminus by two residues (Asn-Met) expressed at the carboxyl terminus of the HNP1 prosegment. Harwig *et al.* identified a minor form of HNP3 in fully differentiated neutrophils with the same Asn-Met dipeptide extension [41] reported here. Thus, CG processing of proHNP1 may also occur *in vivo*.

In contrast to the processing of folded proHNP1 by the azurophilic proteases, misfolded His_6_-proHNP1 was degraded by these enzymes. These results are consistent with studies demonstrating that Cys substitutions in mutants of RMAD4 [32], Crp4 [40] and HD5 [39] are protease sensitive compared to the respective native peptides. However, in contrast to the examples cited above, misfolded His_6_-proHNP1B was disulfide-linked but still susceptible to proteolysis. These data demonstrate that the native disulfide motif is essential for proteolytic stability of the proHNP1 as well as the mature peptide.

Wilson *et al.* demonstrated that proHNP1 could be processed by matrix metalloproteinase 7 (MMP7; matrilysin) to generate a 59-residue molecule with microbicidal activity, but no mature HNP1 (30 amino acids) was detected [42]. Since MMP7 is not present in neutrophils, it was proposed that MMP7 may be involved in the extracellular processing of proHNP1 released from the specific granules of neutrophils. In this regard, Borregaard and co-workers showed that proHNP1 is present in the specific granules of mature human neutrophils and that the propeptide is secreted by activated cells [43]. Since enzymatically active NE is present in inflammatory exudates [44], we hypothesize that proHNP1, elaborated by activated PMNs, may be activated by NE (and possibly other enzymes such as PR3 and CG) in the extracellular milieu. Therefore this process may regulate the extracellular expression of mature α-defensin in the setting of inflammation.

## Materials and Methods

### Expression and Purification of His_6_-proHNP1

The proHNP1 cDNA sequence encoding the natural 75 amino acid residue sequence extended by 5 amino acids (DDDDK; enterokinase recognition site) at the N-terminus was amplified using oligonucleotides (5′-GCGAATTCGATGACGATGACAAGGAGCCACTCCAGGCAAGAGCTG) and (5′-GCGGATCCGGTACCTCGAGTCAGCAGCAGAATGCCCAGAGTC) with plasmid PEC2081 (proHNP1 cloned in pCR2.1 Topo vector) as template. *Eco*RI- and *Xho*I-digested DNA was ligated into pET28a+ vector to generate plasmid PEC2129 which encodes proHNP1 with a 41 amino acid residue N-terminal sequence containing the His_6_-tag (His_6_-proHNP1; Fig. 1). The plasmid was used to transform *Escherichia coli* BL21(DE3)(codon+), generating strain PEC2132-1 which was used for expression and purification of His_6_-proHNP1.

PEC2132-1 cells were cultured for 3 h at 37°C in 3 L of LB+Kanamycin (50 µg/ml) medium and expression of His_6_-proHNP1 was induced by addition of 0.5 mM isopropyl β-D-1-thiogalactopyranoside (IPTG) for an additional 3 h. Cells were harvested by centrifugation and lysed by stirring in 60 ml guanidine lysis buffer (10 mM Tris HCl, 6 M guanidine, 100 mM NaH_2_PO_4_, pH 8.0) at room temperature for 2 h. The lysate was clarified by centrifugation and supernatant was mixed with 6 ml of Ni-resin (His-Bind resin; Novagen) overnight at 4°C. Resin was transferred to a chromatography column, washed with 10 volumes of wash buffer 2 (20 mM Tris HCl, 450 mM NaCl, 0.1% Tween, pH 8.0), and bound proteins were eluted with 3.5 column volumes of elution buffer (20 mM Tris-HCl, 450 mM NaCl, 0.1% Tween, 250 mM imidazole, pH 8.0). The eluate was dialyzed in Spectrapor 3 membrane against 2 L of dialysis buffer (4 M guanidine, 20 mM Tris, pH 8.0), overnight at 4°C.

The dialysate was acidified by addition of acetic acid to 10% (vol/vol) and fractionated on a 10 mm×250 mm Vydac C18 reversed phase high performance liquid chromatography (RP-HPLC) column equilibrated in aqueous 0.1% trifluoroacetic acid (TFA). Proteins were eluted using a water-acetonitrile (ACN) gradient containing 0.1% TFA. As discussed in Results, His_6_-proHNP1 eluted in two peaks, designated peaks A and B with retention times of ∼35 min and 39 min, respectively. Fractions corresponding to Peaks A and B were pooled separately and further purified by C18 RP-HPLC.

### Polyacrylamide gel electrophoresis (PAGE) and western blot analysis

Protein preparations, quantified using the BioRad protein assay (BSA standard) or by measuring absorbance at 280 nm, were analyzed by sodium dodecyl sulfate (SDS)-tricine and acid-urea (AU) gel polyacrylamide gel electrophoresis (PAGE) as described [45]. For western blotting, proteins resolved by SDS-tricine PAGE were transferred to nitrocellulose membranes using a GE Novablot/Pharmacia Biotech Multiphor II system. The membrane was blocked with 5% milk in TTBS (100 mM Tris HCl pH 7.5, 0.9% NaCl, 0.1% Tween 20) and incubated with 0.02 µg/ml anti-His_6_ mouse IgG (Qiagen). The membrane was washed with TTBS and probed with goat anti-Fc mouse IgG-horse radish peroxidase conjugate (1∶20,000 dilution; Sigma) for 1 h, washed with TTBS, and developed by incubation with Supersignal West Pico chemiluminescent substrate (Pierce) and the resulting chemiluminesence was visualized on x-ray film.

### Mass Spectrometry

Matrix assisted laser desorption/ionization time-of-flight mass spectroscopy (MALDI-TOF MS) was employed for analysis of HPLC-purified peptides. Typically, a 0.8 µl sample was mixed 1∶1 with sample matrix (15% w/v α-cyano-4-hydroxycinnamic acid dissolved in 50% ACN+0.1% TFA), spotted and dried on the MALDI-TOF MS sample plate. In some cases, samples were first purified using C18 Zip Tips (Millipore) as recommended by the manufacturer. MALDI-TOF MS was performed on a Microflex LRF MALDI-TOF system (Bruker Daltonics) [46].

### CNBr Cleavage of His_6_-proHNP1

Samples of lyophilized His_6_-proHNP1A (150 µg) or His_6_-proHNP1B (550 µg) were dissolved in 2 ml 80% formic acid containing 20 mg CNBr. The reaction mixtures were sparged with N_2_ and incubated for 18 h in the dark at room temperature after which the solutions were diluted 10-fold with water and lyophilized. The reaction products were dissolved in 0.1% acetic acid and purified by RP-HPLC on an analytical C18 column and analyzed by MALDI-TOF MS. Fractions containing HNP1 were purified further by C18 RP-HPLC.

### Enzymatic treatment of proHNP1

Human NE, PR3, and CG were purchased from Elastin Products Company. Reactions were performed in 50 µl volumes containing 15 µg His_6_-proHNP1 and 2 µg of protease. Buffer for NE and PR3 reactions was 50 mM Tris-HCl pH 7.5, 150 mM NaCl and CG reactions were performed in 50 mM Tris-HCl pH 8.3, 150 mM NaCl [32]. Incubation was performed at 37°C for 18 h after which 5 µl samples were acidified by addition of 10 µl 5% acetic acid, and desalted with C18 Zip Tips (see above) prior to MALDI-TOF MS analysis. The remainder of the reaction mixture was subjected to further purification by RP-HPLC on an analytical C18 column using a 0–60% water-ACN gradient containing 0.1% TFA. The identity of putative HNP-containing species was confirmed by MALDI-TOF MS analysis of reduced (DTT) and alkylated (iodoacetamide) samples which produced a new species with the mass of hexa-S-carboxyamidomethylated peptide derivative (+348 amu) [47].

To generate enzymatically-processed HNP1 for functional studies, 150 µg of His_6_-proHNP1A was digested with 0.1 mg/ml PR3 or CG in 200 µl or with NE in 400 µl reactions for 22 h at 37°C and purified by C18 RP-HPLC as described above.

### Analytical HPLC

HNP1 preparations derived by enzymatic or CNBr treatment were analyzed on an Alliance 2690 Separations Module using a C18 column (3 µm particle, 180 Å pore, 2.0 mm×150 mm, Varian Polaris 5A) with a flow rate of 0.2 ml/min using a 0-to-60% ACN linear gradient in 0.1% aqueous TFA at 1% per minute. Eluting peaks were detected at 210 nm and 280 nm using a Waters 2487 Dual λ absorbance detector using Clarity software. Natural (human neutrophil-derived) HNP1 was used as standard in these analyses.

### Microbicidal assays

The antibacterial activity of HNP1 preparations was evaluated using *Staphylococcus aureus* 502a as the test organism employing a microbicidal assay format described previously [46]. Briefly, log phase cultures of *S. aureus* (∼1×10^4^ CFU/ml) were suspended in 100 µl buffer (20 mM Tris pH 8.0, 28 mM NaCl) plus 0.03% TSB with dilutions of HNP1 ranging from 0 to 8 µg/ml in 0.01% acetic acid for 2 h at 37°C. Aliquots were plated on TSB plates and colonies were counted after overnight incubation at 37°C. Native HNP1 from human leukocytes was used as control. The source of human leukocytes was discarded, de-identified buffy coat preparations from The University of California Irvine-Medical Center clinical labs.

## Supporting Information

Figure S1 **Folding of His_6_-proHNP1B.** His_6_-proHNP1A and His_6_-proHNP1B were analyzed by C18 RP-HPLC and AU-PAGE. Shown are chromatograms and corresponding Coomassie stained gels of His_6_-proHNP1A and His_6_-proHNP1B analyzed **A**) without additional treatment, **B**) after 4 h incubation in TUN buffer alone, and **C**) after 4 h incubation in TUN buffer supplemented with reduced and oxidized glutathione.
(TIF)

Text S1 **Folding of His_6_-proHNP1B in redox buffer system.** In a previous study, Wu *et al.* demonstrated efficient folding of proHNP1 *in vitro* using reduced/oxidized glutathione to mediate thiol-disulfide exchange [29]. Employing this approach, 10 µg of HPLC purified His_6_-proHNP1 was dissolved in 500 µl 50 mM Tris HCl pH 7.5, 0.8 M urea and 100 mM NaCl (TUN buffer) with or without 2 mM reduced and 0.4 mM oxidized glutathione and incubated at 37°C for 4 h at which point the reaction was stopped by addition of 50 µl acetic acid. Reaction products were purified by RP-HPLC on a Vydac analytical C18 column (4.6 mm×250 mm) using a 20-to-55% (1% per min; 1 ml/min flow rate) linear gradient of ACN in 0.1% TFA acetonitrile. Peak fractions were collected and analyzed by AU-PAGE. In the absence of glutathione, the retention time during RP-HPLC and electrophoretic pattern was unchanged compared to the respective starting materials (Fig. S1A and B). After incubation with glutathione, the behavior of proHNP1A was unchanged on C18 RP-HPLC (Fig. S1C) and on AU PAGE (Fig. S1C, lane 5). However, treatment of proHNP1B with glutathione generated a new species that behaved like proHNP1A on both C18 RP-HPLC and AU-PAGE (Fig. S1C). These data indicate that proHNP1A and proHNP1B are folded and misfolded forms of His_6_-proHNP1respectively and that proHNP1B can be folded in the presence of reduced/oxidized glutathione.
(DOC)
