# Supplementary figures and images for: High Fidelity Processing and Activation of the Human α-Defensin HNP1 Precursor by Neutrophil Elastase and Proteinase 3
Source: PLoS One. 2012 Mar 20;7(3):e32469. doi: 10.1371/journal.pone.0032469 (PMC3308943; doi:10.1371/journal.pone.0032469)

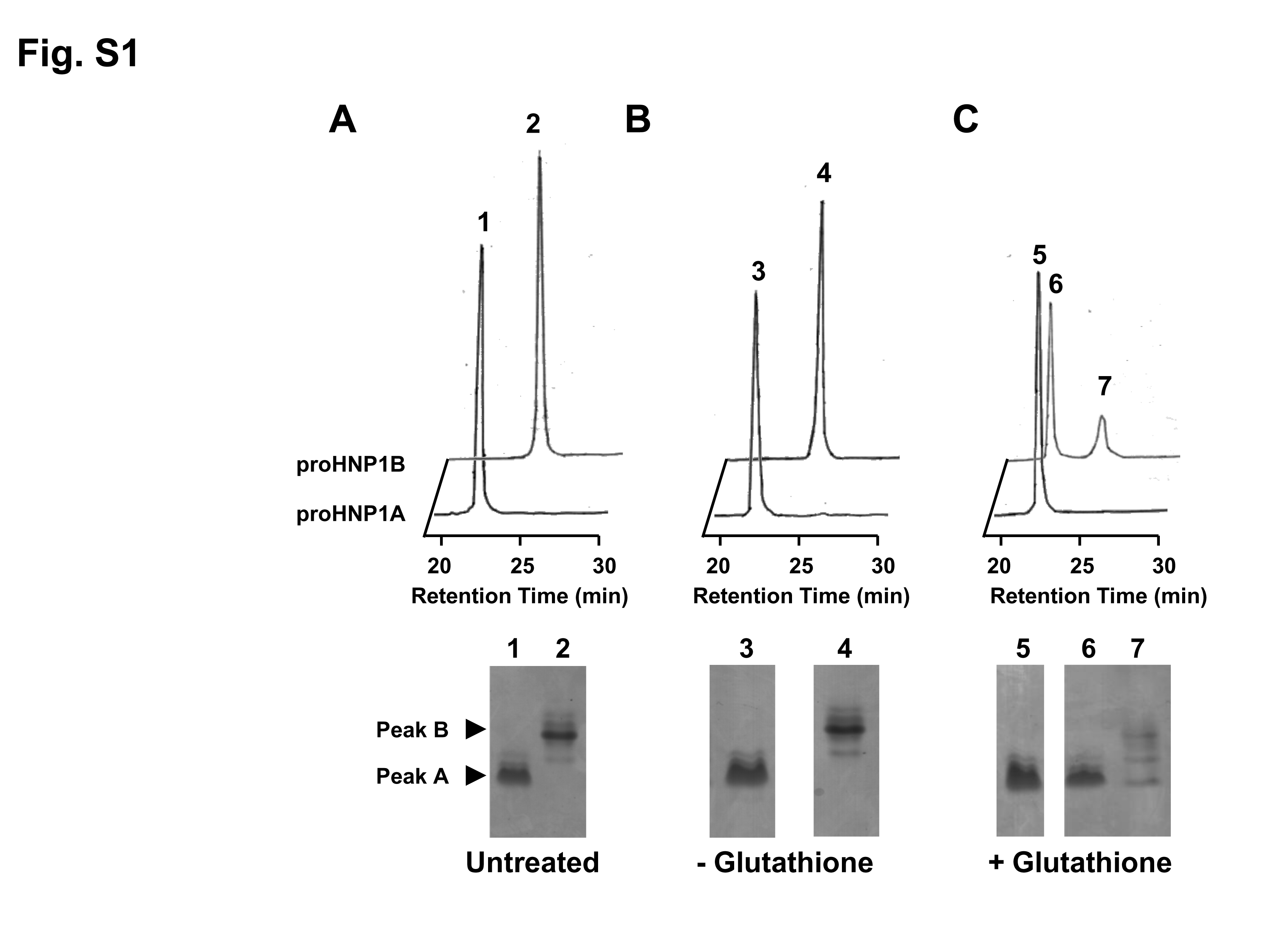

Supplement: Figure S1 — Folding of His6-proHNP1B. His6-proHNP1A and His6-proHNP1B were analyzed by C18 RP-HPLC and AU-PAGE. Shown are chromatograms and corresponding Coomassie stained gels of His6-proHNP1A and His6-proHNP1B analyzed A) without additional treatment, B) after 4 h incubation in TUN buffer alone, and C) after 4 h incubation in TUN buffer supplemented with reduced and oxidized glutathione. (TIF) [file pone.0032469.s001.tif]
