# Supplementary material for: High Fidelity Processing and Activation of the Human α-Defensin HNP1 Precursor by Neutrophil Elastase and Proteinase 3
Source: PLoS One. 2012 Mar 20;7(3):e32469. doi: 10.1371/journal.pone.0032469 (PMC3308943; doi:10.1371/journal.pone.0032469)
Supplement: Text S1 — Folding of His6-proHNP1B in redox buffer system. In a previous study, Wu et al. demonstrated efficient folding of proHNP1 in vitro using reduced/oxidized glutathione to mediate thiol-disulfide exchange [29]. Employing this approach, 10 µg of HPLC purified His6-proHNP1 was dissolved in 500 µl 50 mM Tris HCl pH 7.5, 0.8 M urea and 100 mM NaCl (TUN buffer) with or without 2 mM reduced and 0.4 mM oxidized glutathione and incubated at 37°C for 4 h at which point the reaction was stopped by addition of 50 µl acetic acid. Reaction products were purified by RP-HPLC on a Vydac analytical C18 column (4.6 mm×250 mm) using a 20-to-55% (1% per min; 1 ml/min flow rate) linear gradient of ACN in 0.1% TFA acetonitrile. Peak fractions were collected and analyzed by AU-PAGE. In the absence of glutathione, the retention time during RP-HPLC and electrophoretic pattern was unchanged compared to the respective starting materials (Fig. S1A and B). After incubation with glutathione, the behavior of proHNP1A was unchanged on C18 RP-HPLC (Fig. S1C) and on AU PAGE (Fig. S1C, lane 5). However, treatment of proHNP1B with glutathione generated a new species that behaved like proHNP1A on both C18 RP-HPLC and AU-PAGE (Fig. S1C). These data indicate that proHNP1A and proHNP1B are folded and misfolded forms of His6-proHNP1respectively and that proHNP1B can be folded in the presence of reduced/oxidized glutathione. (DOC) [file pone.0032469.s002.doc]

**High Fidelity Processing and Activation of the Human α-Defensin HNP1**

**Precursor by Neutrophil Elastase and Proteinase 3**

Prasad Tongaonkar^1*^, Amir E. Golji^1^, Patti Tran^1^, André J. Ouellette^1,2^ and Michael E. Selsted^1,2^

^1^Department of Pathology and Laboratory Medicine, Keck School of Medicine, and the ^2^Kenneth Norris Comprehensive Cancer Center, University of Southern California, Los Angeles, California, U.S.A.

**Folding of His_6_-proHNP1B in redox buffer system.**  In a previous study, Wu *et al.* demonstrated efficient folding of proHNP1 *in vitro* using reduced/oxidized glutathione to mediate thiol-disulfide exchange [29]. Employing this approach, 10 μg of HPLC purified His_6_-proHNP1 was dissolved in 500 μl 50 mM Tris HCl pH 7.5, 0.8 M urea and 100 mM NaCl (TUN buffer) with or without 2 mM reduced and 0.4 mM oxidized glutathione and incubated at 37 ^o^C for 4 h at which point the reaction was stopped by addition of 50 μl acetic acid. Reaction products were purified by RP-HPLC on a Vydac analytical C18 column (4.6 mm x 250 mm) using a 20-to-55% (1% per min; 1 ml/min flow rate) linear gradient of ACN in 0.1% TFA acetonitrile. Peak fractions were collected and analyzed by AU-PAGE.

In the absence of glutathione, the retention time during RP-HPLC and electrophoretic pattern was unchanged compared to the respective starting materials (Fig. S1A and B). After incubation with glutathione, the behavior of proHNP1A was unchanged on C18 RP-HPLC (Fig. S1C) and on AU PAGE (Fig. S1C, lane 5). However, treatment of proHNP1B with glutathione generated a new species that behaved like proHNP1A on both C18 RP-HPLC and AU-PAGE (Fig. S1C). These data indicate that proHNP1A and proHNP1B are folded and misfolded forms of His_6_-proHNP1respectively and that proHNP1B can be folded in the presence of reduced/oxidized glutathione.
